# ‘Lemons to lemonade’: Identity integration in researchers with lived experience of psychosis

**DOI:** 10.1111/papt.12582

**Published:** 2025-02-24

**Authors:** Lorna I. Hogg, Alison Branitsky, Anthony P. Morrison, Tim Kurz, Laura G. E. Smith

**Affiliations:** ^1^ Department of Psychology University of Bath Bath UK; ^2^ Oxford Health NHS Foundation Trust Oxford UK; ^3^ Harris Manchester College University of Oxford Oxford UK; ^4^ School of Psychological Sciences University of Manchester Manchester UK; ^5^ Greater Manchester Mental Health NHS Trust Manchester UK; ^6^ School of Psychological Science University of Western Australia Perth Western Australia Australia

**Keywords:** internalised stigma, psychosis, social cure, social identity, stigma, well‐being

## Abstract

**Objectives:**

Lived experience input is becoming recognised as vital to developing and delivering high quality research. However, employment as a lived experience researcher can create identity conflict, which can undermine well‐being. In this study, we explored the nuances of both social identification and identity integration processes in individuals with lived experience of psychosis employed in research.

**Design:**

Qualitative study using a semi‐structured interview format and thematic analyses.

**Methods:**

Fifteen individuals were recruited, all employed in research in UK mental health care trusts or universities. All participants identified as having experience of psychosis and mental health care support and worked as a researcher, in a paid or voluntary capacity.

**Results:**

Two overarching categories were identified in the data, the basis for social identification and the complexity of identity integration within an academic context; specific themes were identified within each of these categories. The data support the value of social identification within this group, although based on shared human experience or being a survivor rather than diagnosis. Challenges to identity integration included conflict between subjectivity and the scientific method and structural stigma. Strong values around using distressing experiences for the benefit of others both furnished self‐growth and connected people in groups. A superordinate identity such as ‘*useful person’* facilitated the integration of lived experience and researcher‐based social identities within the self.

**Conclusions:**

Researching this unique group advances understanding of how social identity forms and functions in a stigmatising context. Findings support the generalisability of the cognitive‐developmental model of social identity integration.

## INTRODUCTION

### The lived experience researcher identity

There are compelling reasons why people with lived experience of a phenomenon under investigation should be involved in researching it. The main reason being to give voice to those who have typically been marginalised within this domain. MAD studies and Critical Survivor eXpertise (CSX), movements that integrate activism and intellectual activity, have sought to position the survivor‐led movement within a broader social and political framework. That is, to challenge current language and assumptions around mental ill health and top‐down power structures, including those associated with research, that effectively devalue and diminish the contribution from those with mental health issues (Beresford, 2019; Clarke et al., 2023; Taggart, 2021). Lived experience researchers are individuals mostly employed either by health services or universities to work in research because of their personal experience of either mental or physical health issues (Bergqvist, 2023). Additional to the ethical drivers, lived experience researchers are likely to have a more accurate and realistic understanding of the phenomenon being researched than those without this experience and the resultant research is likely to be more representative and of higher quality (Oliver et al., 2019). They can draw on personal experience to make research more effective by informing such aspects as study design, choice of measures, participant burden and recruitment and dissemination strategies, alongside potentially destigmatising mental and physical health (Hawke et al., 2022). They may also be particularly skilled at engaging participants in research given shared experience (Staley & Minogue, 2006). Many funding bodies, such as the National Institute of Health Research (NIHR) in the United Kingdom regard this contribution as critical to the research process (NIHR, 2019) and ethical review bodies such as the Integrated Research Application System (IRAS), a necessity for conducting research in the UK National Health Service (NHS), require researchers to report on this contribution. Leading scientific journals such as the Lancet promote lived experience research (Davis et al., 2024) whilst charitable organisations such as the McPin Foundation in the United Kingdom have as their primary focus the advancement of lived experience expertise in research. Thus, lived experience input within research is increasingly regarded as morally irrefutable and critical to the research process.

Although involving people with lived experience in research may bring research advantages, there are potential disadvantages for the researchers themselves (Gupta et al., 2023; Okoroji et al., 2023). Negative aspects can include structural stigma and discrimination in the workplace (Hatzenbuehler, 2016). This is likely to be more pertinent for those with lived experience of mental health issues, given the associated public stigma (Wood et al., 2014). Structural stigma might present as epistemic injustice. Two forms of epistemic injustice have been identified in the literature: testimonial in which an individual's testimony is devalued because they belong to a marginalised group and hermeneutical in which important concepts, including mental ill health, are poorly understood in society because the views of people from marginalised groups have been excluded from relevant discourse (Fricker, 2007; Okoroji et al., 2023). Both of these are relevant to people with psychotic experiences (Harris et al., 2022). Furthermore, there may be incompatibility between the explanatory frameworks of researchers contributing from a professional perspective and those contributing from a lived experience perspective (Faulkner & Thomas, 2002; Rose, 2003). Participant sampling within mental health research is often determined by diagnostic criteria and outcome evaluated in relation to symptom severity or relapse rates, both consistent with the medical model. This can be anathema to those with lived experience who often favour more psychologically or socially informed frameworks (Rose et al., 2011). Continuously drawing on distressing experiences of mental health to inform research practice can have negative personal impacts. Lack of sufficient attention to such issues and associated supportive infrastructure could diminish the contribution of lived experience researchers and potentially place individuals at risk of harm. For the well‐being of such researchers, it is important to clarify roles and expectations and provide appropriate support. This also potentially enables individuals to develop an appropriate research‐related identity and integrate this with a lived experience‐based identity into a coherent and positive sense of self.

The cognitive‐developmental model of social identity integration (CDMSII; Amiot et al., 2015, 2007, 2018) provides a framework for examining identity integration processes. It proposes four stages in the development of social identities within the self: *anticipatory categorisation*, *categorisation*, *compartmentalisation* and *integration*. Within anticipatory categorisation, individuals try to imagine themselves as belonging to a new group. The categorisation stage is characterised by highly differentiated and potentially incompatible identities within which one identity might dominate. Compartmentalisation is a pre‐stage to integration; at this stage, individuals have an awareness of their different social identities and can draw on these simultaneously, but keep them distinct within the self rather than integrated. Full integration reflects a position within which different social identities are assimilated within the self‐concept into a coherent and positive sense of self, and conflict between identities is resolved often through the creation of an overarching identity. Integration is the most advantageous position for health and well‐being (Amiot et al., 2007, 2015, 2018), but may be particularly difficult for those researching mental health from a lived experience perspective.

Recent research addresses the identity implications of working in the field of mental health research as either a lived experience researcher or mental health provider. In a systematic review and narrative synthesis of 13 qualitative papers, Gupta et al. (2023) describe five identity positions reflecting differing levels of integration of lived experience into practice. An integrated identity was considered within the studies reviewed to be the simultaneous holding of, and drawing on, both service user and professional identities. This was found to be quite rare with the more common experience being unintegrated, that is, holding these positions independently. This might represent compartmentalisation within the CDMSII. In this review, identity integration did not address how identities were organised within the self but rather how different identities informed practice. Furthermore, stigma was identified as a driver for lived experience work, that is, working in this capacity offered an opportunity to move beyond the stigmatised service user identity. The question of whether and how social identities can form in the context of stigma is important to consider in relation to understanding the lived experience researcher role.

### Social identity and stigma

Social identity theory (SIT) provides a helpful lens through which to consider issues of social identity and stigma for lived experience researchers (Tajfel & Turner, 1979). Within SIT, social identity refers to that sense of self comprised of a sense of belonging in social groups. SIT proposes that individuals are motivated to join social groups that enhance their self‐esteem. This can present challenges for people with lived mental health experience who may not feel motivated to belong to social groups comprising others with similar experiences, given the associated stigma (Kular et al., 2019; Rüsch et al., 2013).

The question of whether social identification can develop in the context of stigma is important. The social identity and health hypotheses (SIAH) propose that social identities can be both positive and negative for well‐being, depending on how they are perceived (Haslam et al., 2018). This suggests that identifying with stigmatised groups could be harmful for health; however, evidence is mixed (Veelen et al., 2020), with some suggesting that such an identity might operate as a social curse and block opportunities for social support (Kellezi et al., 2019) as opposed to the social cure suggested by the SIAH hypotheses (Haslam et al., 2018). Other research suggests that concealing a stigmatised identity can be self‐defeating in undermining authenticity and a coherent sense of self (Crabtree & Pillow, 2020). Furthermore, the rejection–identification model (RIM) of Branscombe and colleagues suggests that identification with others in a minoritised group can be protective of well‐being in the context of discrimination (Branscombe et al., 1999; Jetten et al., 2009). Thus, developing a positive social identity, such as someone with mental health challenges, can be problematic, though not impossible, and might even be protective within a stigmatising context. A focus on researchers with lived experience of psychosis is particularly helpful in exploring social identification and integration processes given the need to identify at some level as having an identity associated with their lived experience, and given that psychosis is one of the most stigmatised of mental health conditions (Ahmed et al., 2020; Wood et al., 2014).

### Psychosis, social identity and identity integration

*Psychosis* is an umbrella term that reflects a mix of experiences that include some combination of voice‐hearing, beliefs not shared by others and disruption to thinking, affect and behaviour (American Psychiatric Association, 2022; World Health Organization, 1992). Due to the variability in experiences and the fact that psychotic experiences lie on a continuum with *normal* experiences, the diagnosis is contested (Bentall, 2004; Read et al., 2004). Many people do not identify with a psychosis diagnosis because of associated stigma and prefer a more experience‐centric identity such as *voice hearer*.

Recent research attests to the value of social identification and integration processes for well‐being in people who hear voices. Hogg et al. (2023) examined the cross‐sectional association and predictive power of a social identity associated with hearing voices. Both a voice hearer social identity and the integration of this identity with other important social identities were found to be associated with well‐being with integration accounting for variance in the relationship beyond that of social identity and further predicting well‐being after 6 months. Perception of empathy in other voice hearers was associated with the development of a voice‐hearing social identity, and perception of empathy in those who do not themselves hear voices was associated with the integration of a voice‐hearing identity. Extrapolating from this work to lived experience researchers, it might be predicted that empathic encounters with other lived experience researchers, for example in the context of a peer support group, will foster a positive sense of self as a lived experience researcher. Furthermore, perceiving work colleagues who do not themselves have psychotic experiences to be positively disposed, as opposed to stigmatising, towards people with psychosis, will likely facilitate the integration of lived experience and researcher‐based social identities.

### The current study

Given the developing evidence for the existence and benefit of a social identity connected to psychotic experiences (Hogg et al., 2022, 2023; Sheaves et al., 2021), this study uses a qualitative methodology to explore the subtleties around the formation of such an identity and its perceived impact on well‐being. A further objective is to build on the work of Gupta et al. (2023) to uncover the richness surrounding the identity integration process within the self by focusing on individuals employed in research based on their personal experience of psychosis. This participant group allows the opportunity to investigate both identification and integration processes. Participants will have a social identity based on their mental health experience given this is the basis for their employment in the role. In this study, we specifically selected for experience of psychosis given it is one of the most stigmatising mental health conditions and we wanted to explore factors important to the formation of a stigmatising social identity together with the integration of this with other important social identities into self‐concept. Participants will also have an identity as a researcher. The lived experience researcher role requires some degree of integration of these two social identities (i.e. that based on the experience of psychosis and that based on the researcher role). These social identities are potentially conflicting, and both might also be highly nuanced (Davis et al., 2024; Hawke et al., 2022; Richmond et al., 2023).

## METHODS

### Participants

Fifteen participants were recruited, all of whom at the time of participation identified as employed as researchers in UK universities or NHS Mental Healthcare Trusts based on their lived experience of psychosis. Such participants were considered likely to have high contact with others experiencing psychosis and insight into the concepts under investigation, namely social identification. Participants also needed to have experience of receiving a psychosis diagnosis and mental health care support in relation to this diagnosis, according to self‐report. Self‐reported diagnosis was considered sufficient given the study concerned social identity processes. Additionally, participants needed to be aged over 18 years, able to engage in an interview conducted in English, able to consent to participate and not too distressed to be adversely affected by discussing their lived experience. Participants were recruited through UK University psychosis research groups and charities, such as The McPin Foundation, and research networks, including the Recovery Research Network.

The sample reflected a mix of ethnicities, ages and genders. Complete socio‐demographic and clinical information is detailed in Table 1. Most participants were still having psychotic experiences at the time of participating (60%), and taking prescribed medication for psychosis (60%). Diagnoses included schizophrenia (33.3%), psychosis (13.3%), post‐traumatic stress disorder (6.7%) and bipolar disorder (6.7%). Six participants (40%) reported receiving multiple diagnoses over time, including psychosis.

**TABLE 1:** Socio‐demographic and clinical characteristics.

Variable	Frequency (*N* = 15) *N* (%)
Age	
Mean	39.7 years
Median	39.0 years
Range	26–67 years
SD	10.2
Gender	
Male	7 (46.7%)
Female	7 (46.7%)
Prefer not to say	1 (6.7%)
Ethnicity	
White British	6 (40%)
White Other	1 (6.7%)
Indian	3 (20%)
Black African	4 (26.7%)
Black Other	1 (6.7%)
Diagnosis	
Schizophrenia	5 (33.3%)
Psychosis	2 (13.3%)
Post‐traumatic stress disorder	1 (6.7%)
Bipolar disorder	1 (6.7%)
Multiple diagnoses including psychosis	6 (40%)
Employer	
UK university	3 (20%)
NHS mental healthcare trust	9 (60%)
University + NHS Trust	2 (13.3%)
University, NHS Trust + Charity	1 (6.7%)
Hours worked per week as lived experience researcher (*N* = 14)	
Median	18.8 h
Range	2–37.5 h
SD	15.0
Length of time worked as lived experience researcher	
Median	4.00 years
Range	1–12 years
SD	4.0
Time since psychosis started	
Median	12.0 years
Range	3–34 years
SD	10.1
Psychosis continuing	
Yes	9 (60%)
No	6 (40%)
Currently taking prescribed medication	
Yes	9 (60%)
No	6 (40%)

### Materials and procedure

Ethical approval was obtained from a UK university research ethics committee (approval number 21‐266). Potential participants responded to posters and adverts on organisational websites. They were then sent participant information, a consent form and socio‐demographic and clinical questionnaire via email. They were given minimum 48 hours to consider these before being contacted by telephone to explain the study and answer any questions. All participants then completed the consent form and were booked for interview.

A semistructured interview schedule (see Data S1) was developed and refined through piloting between the lead author and a co‐author, who is a researcher with lived experience of psychosis. Participants were first provided with a definition of the concept of social identity as that aspect of self‐concept that relates to a felt sense of belonging in social groups and shapes attitudes and behaviour. Examples of social groups were shared including those based on sport, religion, nationality, occupation, sexual orientation, ethnic group, gender and health condition. Participants were then asked about the extent to which they considered themselves to have a social identity connected to their lived experience of psychosis and if so, what the basis for this social identity was. Further interview topics included: the amount of contact with others with lived experience of psychosis, the norms and values of the social group, positives and negatives of identifying as a member of this social group, similarity with others in the group, the centrality of this identity to sense of self and the integration of this social identity with other important social identities, including specifically that of researcher/academic.

Interviews lasted 30–90 min and recruitment took place from August 2022 to August 2023. All interviews were conducted online using the Microsoft Teams platform (MS Teams) by the lead author, a clinical psychologist and researcher specialising in psychosis. Verbatim interview content was captured using the transcription function within MS Teams. Audio recordings were also made simultaneously using a dictaphone to provide back‐ups. Transcriptions were clarified and refined by the lead author, lived experience author and a doctoral student. Participant names and any other identifying information were pseudonymised. Participant numbers are provided to differentiate each anonymised quote. All other contextual information has been excluded to protect confidentiality given participants were drawn from a relatively small pool. All participants were emailed a debrief sheet on completion of the study detailing sources of support if needed. Participants were paid £15 for their contribution to the study.

As a validity check, all participants were given the option of either attending a focus group to consider the draft analyses and implications or contribute via email (to protect confidentiality). Four participants attended the focus group and a further two contributed via email. Participants endorsed themes with some further refinement. The final analysis reflects this feedback.

### Analytic strategy

Data were analysed using thematic analyses (Braun & Clarke, 2006, 2021), facilitated by NVivo 12 software (Lumivero, 2017). A symbolic interactionist epistemological approach was taken to the data with a view to understanding how participants perceived and attached meaning to their experiences. Familiarisation with the data involved the lead author listening to interview recordings and repeatedly reading transcripts to generate initial codes inductively. Codes relating to research questions were then grouped to generate initial themes and subthemes. Themes and example quotes were next reviewed for credibility by all co‐authors. Finally, participants in the study reviewed the validity of the themes and sample quotes either via a focus group or individually via email. Themes were refined through this process to produce the final thematic map. Additional points made as part of this validation process are noted as they appear in the Results section.

## RESULTS

**FIGURE 1:**
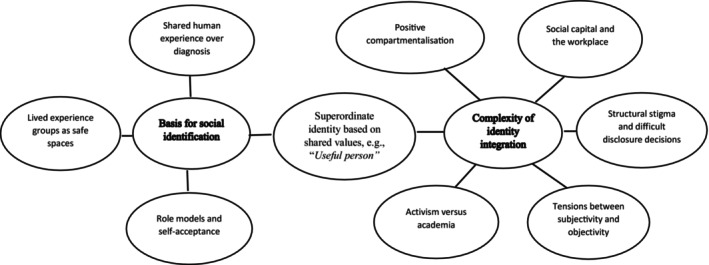
Thematic map of social identification processes for researchers with lived experience of psychosis.

Specific themes identified within the data were organised into two overarching categories: (1) The basis for social identification and (2) the complexity of identity integration within an academic context. We first introduce the general nature of these two categories here, before providing fuller elaboration and empirical explication of them in the sections that follow. Figure 1 depicts categories and associated themes.

In relation to Category 1, *the basis for social identification*, all participants were able to identify a social group related to their mental health experiences to which they felt a sense of belonging. However, as can be seen in Figure 1, the specific social identities varied; perceived shared human experiences such as *voice hearer* or *survivor* of trauma or treatment within mental health services were preferred over a shared identity based on diagnoses. The sense of belonging to these groups met the criteria expected of a social identity; they provided individuals with collective self‐esteem, a shared sense of purpose, shared values and a safe supportive space where they felt able to be open and honest about their mental health experiences without fear of judgement or rejection. Important to the forming of such social identities was inspiration from charismatic leaders or role models. These were individuals prototypical of the group, with similar mental health experiences, often involved in activism, who engendered hope and portrayed a positive sense of self as someone with mental health problems. Thus, the data supported the existence of a social identity connected to the experience of psychosis in lived experience researchers; however, the basis for this identity was nuanced and not based on diagnostic categories.

In relation to Category 2, *the complexity of identity integration within an academic context*; it is clear from the data that being able to integrate a lived experience‐based social identity with that of researcher or academic is challenging. The ‘objective’ and ‘scientific’ nature of academic endeavour does not fit comfortably with knowledge based on subjective experience. Lived experience researchers often enter research from a background in activism and view academia as a more powerful position from which to influence attitudes and bring about change, given that it has been constructed as having higher status. However, given experiences of structural stigma (Gupta et al., 2023) and the epistemic injustice that people with unusual perceptual experiences endure (Harris et al., 2022), the reality is often that they feel their influence is diminished. This is not universally true, and positive examples of allyship are reported. Participants reported that one factor that makes lived experience research feel worthwhile and also facilitates identity integration is holding a superordinate social identity such as that of ‘*useful person’*, in the words of one participant. This is reflected in a shared commitment to making the world a better place and acting collectively to improve outcomes for those with similar mental health problems. As can be seen from Figure 1, this social identity of ‘*useful person’* both connects people in groups and provides a superordinate identity within which lived experience and researcher‐based social identities can be integrated within the individual into a coherent and positive sense of self.

### The basis for social identification

#### The connecting power of shared human experience over diagnosis

Participants in this study valued a shared social identity related to their psychosis. However, the basis for this varied between individuals and was generally not premised on diagnosis, which was not perceived as providing anything of social psychological value:

And genuinely for about five years it was “X the schizophrenic”. That was my identity. And that was f*****g sad. I don't want to go back there. (…) I didn't even notice that was my identity until I no longer was identifying that way. (P5)

This may reflect the fact that diagnosis is an externally given label, often based on an unwanted medicalisation of experiences. This finding is consistent with other research in the field of marginalised identities. In a study by Best et al. (2018) on recovery in people with drug and alcohol misuse issues, a social identity based on recovery was found to be superior to one based on illness in terms of recovery outcomes. A similar phenomenon has been reported in relation to online support groups for people with eating disorders (McNamara & Parsons, 2016). An implication of this finding is that clinicians should give careful thought to the personal and social impacts of diagnosis (Angermeyer & Matschinger, 2003; Lersner et al., 2019; Rosenfield, 1997; Vass et al., 2017), particularly given challenges to the validity and reliability of some diagnoses, such as that of psychosis (Read et al., 2004).

In our study, social identification was more often reported as based on having experiences in common and there was variation in what this meant between individuals. For some, it was having more general mental health problems in common. Through sharing a social identity, participants appeared to be better able to provide support to each other, consistent with the social identity/self‐categorisation model of stress (Haslam et al., 2005):

So, my identity is a person with mental health problems, definitely, and I feel a kinship there. I also feel I have some friends who are very close friends because of that but we don't share the same diagnosis, each of us. It's more that we share symptoms and therefore are able to support each other within that. I think because none of the three of us that I'm thinking about now really lean too heavily into the diagnosis kind of side of things, that enables us then to put that aside and, like, what are you dealing with in life right now? How can we support each other? (P11)

For others, social identity was more accurately defined in terms of a *survivor* identity, which is survivor of trauma. The trauma might be early abusive experiences that led to the initial development of mental health problems but could also include the trauma of treatment within mental health services:

I think just the survivor community even though it would be filled with people the vast majority who would have had a diagnosis of psychosis or schizophrenia but I think we saw ourselves as survivors of abuse, survivors of psychiatry. (…) For some, the onus of their trauma was actually less what had happened to them pre‐mental health problems and more on how the system had medicalised and further re‐traumatised them and made their recoveries more difficult. Um so I think survivors of adversities of multiple types and descriptions. (P3)

A *survivor* identity might be viewed as an example of a commonly reported strategy for coping with social identity‐based threats, namely changing the definition of the group to something more positive (Butera & Levine, 2009). The existence of a distinct social identity associated with the experience of psychosis for lived experience researchers adds to the weight of evidence in support of the value of social identification even within stigmatised minority groups as suggested by the rejection‐identification model (Branscombe et al., 1999). The social identities described within our study appeared to be positive for well‐being, and to enhance self‐esteem and make it more possible to feel confident in contributing within a research context. This is consistent with the social identity and health hypotheses (Haslam et al., 2018), and the previous work of Hogg et al. (2022) and Sheaves et al. (2021) on social identification in voice hearers.

#### Lived experience social groups as safe spaces

Fundamental to the forming of social identities was experiencing social groups, online or in‐person, as providing a *safe space* where individuals would be free from criticism, judgement or discrimination and where mental health experiences were validated and normalised. This was hugely valued given the stigmatising and marginalising nature of the identity:

I was regularly going to these meetings (with other mental health service users), meeting other people who have been in similar situations. Really, you know like really making friendships and, you know, being around people who understood what I had gone through. (…) Going to these groups, you really have this sense of safety. It was like a safe space for connection and yeah, it was very beneficial. (P10)

Relational processes may be mechanisms that connect social identification to well‐being. In previous research, in‐group empathy was associated with social identification and well‐being in voice hearers (Hogg et al., 2023). This process through which small group discussion and supportive interactions shape social identity processes has also been documented by other researchers (Postmes et al., 2005).

The confidence that lived experience‐based social groups engendered was contrasted with interactions with clinicians or other researchers who could be clumsy in their handling of mental health issues:

I've worked with clinicians who are really wary about, you know, how do I broach this subject? How do I talk about this? And you know, I've worked with researchers who are really wary about talking about things for fear of this, that, or the other. Whereas when you're with a group of people who have experienced that or had similar experiences, actually, there's a freedom to be open and honest and not worry about that. (P6)

It is clear that participants in our study felt that lived experience‐based social groups provided social support and fostered a sense of solidarity and belonging within the group, which was important to social identification. This reflects the self‐investment component of the multicomponent model of social identification (Leach et al., 2008). Taken together with the findings regarding diagnosis above, this work suggests that, for those with psychosis, self‐investment is a more important basis for group belonging that self‐definition. That is the well‐being benefits were derived from the support experienced within the group rather than feeling defined by the group or homogeneity within the group.

#### Role models as facilitators of self‐acceptance

Participants reported looking to their group memberships for hopefulness and inspiration or support to develop self‐acceptance and a stronger sense of self:

When you actually meet people that have experienced it (psychosis), it's quite eye‐opening. Well, it was eye‐opening for me to kind of get a sense that these are kind of ordinary people who experience different things and they've found a place of acceptance and I think it helps me to kind of find my own kind of acceptance with it all really. (P9)

Role models were seen as critical both in the cementing of social bonds but also inspiring radical acceptance of the challenges of living with a serious mental health condition. Role models were perceived to promote personal growth in others by embodying a positive sense of self as someone who experiences psychosis:

I think having had that community of fellow survivors who'd instilled in me very early on that this was not something to feel ashamed of. And seeing their example, seeing their like sort of their courage. And, you know, just really admirable people like the sort of people that you would really enjoy being in their company. They were really likable. They're really smart and inspiring. And their example of not being ashamed sort of made me sort of think, well, I wanna be like them, I don't wanna sort of be ashamed of it. I think those kind of like role models really and that kind of internalised sense of pride and positive identity. (P3)

Thus, role models—charismatic individuals with similar mental health challenges who had publicly disclosed their own psychosis—were perceived as both relatable and worthy of emulating. Such role models facilitated social identification, self‐esteem and personal growth in others and motivation to work collectively to bring about social change. This is consistent with recent advances in the social identity theory of leadership which suggests that leaders who reflect idealised prototypes are revered (Steffens et al., 2021). The point was also made in the current study, as part of the validation process, that the increased self‐acceptance that such role models, and also peer support groups, gave individuals made it possible for people to feel confident enough to work in an academic context.

### The complexity of identity integration

#### Activism versus academia

Many lived experience researchers reported being drawn to research as an avenue for activism. They believed that a role in research would give them agency and a platform to advance the interests of people who experience mental health challenges. This resonates with the growing interest in scholar activism (Quaye et al., 2017) and MAD activism in particular (Beresford, 2019):

(…) by speaking to other people through my work, I hope that it can help reduce stigma and help people to think it's okay to talk about it. (P2)

So, I tend to not be out on the streets waving placards. I'd rather kind of be within different systems advocating for change, you know, more meaningfully for what I want to do, I would say. (P9)

However, for some, there were unanticipated consequences of the researcher role in that they felt they had to tone down their message:

The message remains quite consistent but it's much more I guess for want of a better word, more inhibited by my professional role where I feel I have to be more cautious about what I say. (P3)

One feared consequence was being perceived to be self‐serving:

So people who don't work in research and people who don't work clinically who have experience of psychosis and, you know, they might do advocacy work on social media or be influencers or whatever. And then obviously me being a lived experience researcher, I don't know how much I can engage in their advocacy work and, you know, also do that myself because I might be perceived to be promoting an agenda for my own research… (P8)

The restrictions imposed by the lived experience researcher identity were reported by some to result in a loss of felt authenticity at a personal level and disconnectedness, rather than integration of the lived experience and researcher identities:

I've sort of become more professionalised in terms of how I talk about things so that raw emotion, that, you know, that authentic experience that might come from other people with lived experience, I don't feel that comes across anymore. (P8)

While participants reported being attracted to academia to advance an activist agenda, there were incompatibilities between these two social identities of activist and researcher that created tension for individuals. This challenge of integrating different social identities within a work context, extended to other marginalised identities such as those based on ethnicity or neurodiversity. Compatibility between the various social identities of lived experience researchers appeared to be important to developing an integrated sense of self and related well‐being. This is important given evidence that identity compatibility is protective of health and well‐being particularly through important life changes (Jetten et al., 2009).

#### Tensions between subjectivity and objectivity

The lived experience researcher role is premised on drawing on very subjective and often sensitive experiences to enrich research endeavours. Research, by contrast, typically adheres to a more objective scientific method involving systematic observation, measurement and experimentation. Participants spoke of uncertainty over when and how to bring their lived experience into such a process. The point was made, in the validation process, that a lived experience perspective might lend itself better to qualitative over quantitative methodologies, although this would depend on the specific knowledge, skills and experience of the individual. Navigating this disparity between subjective and objective can present challenges for both lived experience researchers and their colleagues, not least how to integrate these differing knowledge bases. This is critical given the nature of the lived experience researcher role. One participant spoke of being asked by a manager to effectively downplay their lived experience:

…knowing when to draw on what at what point and I think with my new, you know, employer, I've discussed that and they've said you're going to need to wear different hats at different times. Umm. And they did actually say (…) “We do want you to draw on your researcher side more than your lived experience side” (P8)

Thus, as depicted in Figure 1, there are a range of challenges that lived experience researchers need to navigate to make a useful contribution to research. The workplace often feels like a *brave space* (Arao & Clemens, 2013), rather *safe space* where individuals have to consider carefully how to bring in their lived experience and at times manage colleagues' clumsy efforts to understand their role and relate.

#### Structural stigma and difficult disclosure decisions

Perceptions of outgroup empathy have been identified in previous research as associated with the integration of a psychosis social identity with other important social identities for individuals (Hogg et al., 2023). By contrast, in the current study, some participants spoke of fear of their behaviour being misinterpreted as not coping given their mental health. One participant relayed a distressing experience of their behaviour being misconstrued as illness, fuelling a sense of being *othered*:

I feel I maybe do come across as a bit odd, or unusual. Like so my clinical supervisor's just like saying how he feels I jump around a lot. And I just felt he was getting very close to like pathologising me (…) But I'd say if you asked me, I could have explained the link. But he just assumed I was being weird and broken (…) it just made me feel a bit othered. (P5)

Despite being employed on the basis of their lived experience, participants in these contexts reported anxiety and difficulty in making decisions about what to disclose of their personal experience for fear of judgement:

I'd probably kind of suss them out first and kind of think “How are they going to react?” And if I sort of think, if I think that they'd be nice and not judge me too much, then I'd probably tell them about it. (P2)

It is clear that lived experience researchers at times felt duped in entering a work environment that had not considered carefully how to embrace the unique perspective that they could contribute, and use this to enhance the quality of research. Furthermore, at times the attitudes and prejudices of other colleagues without disclosed lived experience led lived experience researchers to feel anxious and insecure, which led to secrecy and fear of disclosure. This context is less likely to facilitate integration of identities into self‐concept and improved well‐being.

#### Social capital and the workplace

There was clear diversity in work environments and many participants spoke of supportive empathic managers, supervisors and research colleagues, including in relation to managing mental health challenges. Such interventions could be specific to individuals, but some participants spoke of working in supportive teams where everyone, whether they had declared mental health needs or not, was treated respectfully and equitably:

It's just this real sense of, certainly from my perspective, just this real sense of equity, yes and purpose. Umm, you know everybody's valued for their different contributions. (…) It really just embodies this culture of support and openness, and lack of judgment. (P3)

Factors that contributed to a feeling of safety and better identity integration included opinions being encouraged and respected:

They were lovely in that they very much let me have a voice and made me feel like I was part of things other than an add on or a token kind of person in the room. (P11)

Participants had interesting suggestions for ways that teams could better embrace lived experience researchers and cultivate a greater sense of social safety in the workplace. These included allyship from colleagues, opportunities for co‐working and good leadership (i.e. leaders who epitomised and enacted the shared values and purpose of the research group, including treating all group members as equals). Induction processes were considered critical, and it was suggested that initial ‘getting to know you’ conversations would help to clarify expectations in both directions, understand relevant lived experience and consider how lived experience might augment the work of the team. Regular check‐ins were suggested with opportunity to reflect on the personal impacts of the work, provide prompt non‐judgmental support with mental health needs when these are current and provide support around difficult tasks such as presentations. The point was made that people who have experienced trauma might be more sensitive to power imbalances and may have greater need for those in power to demonstrate their collegiality; trauma‐informed supervision was also suggested as a potentially helpful approach for all research staff, not just lived experience researchers. Given experience of challenges in navigating the workplace environment, lived experience researchers are ideally positioned to advise research teams on how best to embrace their contribution. This, in turn, is empowering and potentially helpful in enabling lived experience researchers to integrate their lived experience‐based identity with that of researcher.

#### Positive compartmentalisation

Within the cognitive‐developmental model of social identity integration (CDMSII) *compartmentalisation* is conceptualised as a pre‐stage to identity *integration* within which social identities are kept separate and are context dependent (Amiot et al., 2007, 2015). However, in this study, a few participants who appeared to have highly integrated identities within the workplace spoke of the need to find a balance such that they could enact non‐psychosis‐related identities without these being overshadowed by psychosis (i.e. to compartmentalise the identities).

It may be that people do not necessarily follow the CDMSII stages sequentially and it may be a functional strategy to choose to compartmentalise, even for people with integrated identities. Some participants felt that they had found balance between their social identities through lived experience work, that is, they had moved from a position of *identity engulfment* (Konsztowicz, 2019) or *categorisation* within the CDMSII model early in the development of their psychosis in which their identity as someone with psychosis was all‐consuming in an unhelpful way, to a more balanced and better integrated position. One participant spoke of using a pseudonym on Facebook in order to have a space where they could get some respite from their identity as someone with psychosis:

I find that a helpful thing to have – a place to go to where I never have to talk about that, you know, whether I'm struggling or how I'm coping with things or having to be any kind of, you know, ambassador for like how to treat people well in mental health or how to deal with symptoms or how I'm dealing with things or talking about diagnostic identities. No, there's none of that. It's just a space where I can let all that go and just be. That is a helpful thing. (P11)

Thus, while being able to be open and honest about personal experience of psychosis is healthy in terms of a coherent sense of self, it is also challenging in having to live that life all the time. It was very clear that working in a research context that perpetually triggers difficult and distressing personal experiences is hugely challenging and participants needed to develop strategies for managing this. One participant spoke of working hard to desensitise themselves by repeatedly rehearsing what they plan to say at home on their own until they can say it without feeling distressed. Participants also spoke of the need to know their own personal limits and to feel able to say to colleagues when they do not feel able to talk about particular aspects of their lived experience. Such boundaries are clearly important in terms of creating safe spaces in the workplace for people. Having time‐out and spaces where other identities can flourish was also reported to be important for well‐being.

#### A superordinate identity based on shared values

One way of integrating identities within the self, and potentially resolving conflict between identities, is to develop a superordinate identity that binds different social identities into a coherent whole (Amiot et al., 2015). A strong motivation for engaging in lived experience research reported by participants was the use of distressing personal experience for good, namely, to benefit others going through similar experiences. One participant (P4) described this as turning ‘*lemons to lemonade’*. This attitude connects with the strong activist routes of some and was a key motivation in the forming of social identities based on lived experience. As one participant put it:

You know, I guess my broadest social identity is someone who is useful. (P4)

People sought out like‐minded individuals who had similar mental health experiences to work together in the form of collective action for the betterment of others in the wider disadvantaged social group. Thus, as depicted in Figure 1, and consistent with CDMSII, shared values and purpose not only connected individuals to groups but also facilitated the formation of a superordinate identity that facilitated the integration of lived experience and researcher identities:

It does feel like just this big communal team. Where our sort of shared identity is that we want to make things better in the mental health system. You know, that's what motivates all of us. (P3)

In the words of another lived experience researcher:

In an ideal world (…) you would hope that there wouldn't have to be a researcher with a big banner above their head saying ‘mental’ in the room. You know, anybody with any lived experience of any kind should feel free to share (…) Wouldn't it be better if people could just share stuff that would be advantageous to the running of a trial without having to have a big label on them that, you know, permits them to talk from this perspective? (P11)

These findings highlight how complex and challenging it can be to integrate a social identity based on lived experience of psychosis with that based on research. They reflect the lack of clarity around the lived experience researcher role within some contexts, power differentials and ongoing threat associated with structural stigma. Those seeking such roles are often motivated by a higher order desire to use their lived experience to make a difference to the lives of others. The ensuing renewed sense of purpose together with strengthened relationships and self‐concept inherent within this process have been described by other researchers as key elements of post‐traumatic growth in the aftermath of psychosis (Jordan et al. 2020, 2023; Slade et al., 2019). Creating research environments that support the development of such a superordinate identity hold the promise of supporting identity integration for lived experience researchers and others in the research group bringing different experience and social identities to the research endeavour.

## DISCUSSION

This study advances understanding of the nuances involved in social identification and identity integration in the context of stigma. Despite the stigmatising nature of psychosis, lived experience researchers reported valuing a shared identity based broadly on psychotic experiences. Our research highlights that the basis for this social identity is crucial if it is to be meaningful. Diagnosis was not considered to be a helpful basis for social identification. Shared human experience such as having specific psychotic experiences in common, or similar histories of surviving trauma including trauma arising from treatment within mental health services, was the connecting force.

Furthermore, our research elaborates the process of identity integration within the self for those with a dual identity as someone with lived experience and also a researcher. The shared social identity of ‘*useful person’ (as described by one participant)* and the motivation to use lived experience in collective action for the betterment of others connected people within social groups and also facilitated identity integration and self‐cohesion across identities including other intersecting identities such as that of activist. This is important given the relationship between identity integration and well‐being (Amiot et al., 2007, 2015, 2018). It indicates that Amiot's cognitive‐developmental model of social identity integration (CDMSII) developed in relation to the integration of cultural identities is also applicable in this more specific lived experience researcher context. A further interesting finding in this research is the partial compartmentalisation of a lived experience‐based social identity. This adds novelty by advancing the CDMSII indicating that even in the context of a well‐integrated social identity, in the context of stigma, individuals prefer to retain choice over when and where this identity is enacted.

In both lived experience and research, and also activism and research, it is the circumstances that lead to people being in the role that can then create the conflict. Our research indicates that it is not sufficient to just appoint people to lived experience research roles; more consideration is needed to help people to integrate their identities in this context. A superordinate identity as someone who helps others not only provides integration across these social identities but also this in turn brings meaning to lived experience and supports personal growth. Role models, that is, charismatic others who represent *ideal‐type prototypes*, who speak out about their own similar lived experience are valued in this process of self‐acceptance and growth. Stigma and discrimination in the workplace and also allyship are powerful influences on the extent to which lived experience researchers can be their authentic selves and make a valid contribution.

The importance of involving people with lived experience in mental health research extends beyond the social–psychological needs of the individual. Consistent with MAD studies, this work needs to be considered within a broader social and political context (Beresford, 2019; Clarke et al., 2023; Taggart, 2021). The knowledge of people who have mental health experiences has traditionally been invalidated, in part due to assumptions about individuals' inability to reason due to mental ill health, or epistemic injustice (Fricker, 2007; Harris et al., 2022; Okoroji et al., 2023), and further because their perspective might contradict the position of those in power. As in the current study, objectivity within academia can lead to the subjugation of experiential knowledge. To bring about much needed change, it is imperative that those in positions of power move beyond a tokenistic involvement of people with lived mental health experience to essentially rubber‐stamp their own actions and address the inequities within research to create openings for those with lived experience within power and leadership structures (Jones et al., 2020; Scholz et al., 2019). This gives rise to questions around the true purpose of inclusion within research and how we conceptualise mental health experience. It is important that conceptualisations are dynamic and reflect the changing understanding of mental health over time. Also, that they take account of individuals' differing experiences of mental ill health and differing explanatory frameworks (e.g. medical and social–psychological). Furthermore, many individuals within this community will have multiple overlapping stigmatised social identities, such as neurodiversity, race, gender and class. It is important that power structures within research recognise this complexity.

### Limitations

Our study was conducted with a relatively homogeneous group of people with psychosis in that all participants, at the time of recruitment, were working in UK healthcare and universities in a lived experience research capacity. This selection approach was important to investigate the phenomena under consideration; however, the results may not be generalisable to identity integration for others, including others with lived experience of psychosis.

The size of the sample was restricted by the very specific nature of the role and current limited expansion of such diversity within research. The sample included both academics with disclosed lived experience of mental health and those advising on research from a service user perspective. Given the overall size of the sample, it was not possible to separate out the respective contributions of these groups. The analyses focused on common themes across the data set. However, it will be important for future research to explore the differing work‐based social identities and basis for identity integration in these groups. It may be that those with an academic track record find it easier to develop a strong work‐based social identity and meet fewer challenges to integrating this with a mental health‐related social identity, but this remains to be investigated.

### Future directions

The findings of this research generate some testable hypotheses regarding factors that might facilitate social identification for people with psychosis. These include the perception of shared human experience, similar values and purpose and access to safe spaces with similar others. Given that lived experience researchers are often working in teams where they represent the sole contributor from this perspective, there is scope for establishing peer support networks or educational programmes that extend *across* different research groups. This point was made by one participant in the validation process. The study also suggests that interventions that promote identity integration, specifically the blending of subjective experience with a scientific approach to research and also activism with research, will be important to well‐being in this context. Our research would suggest that role models are important in this respect. Their contribution could be tested in experimental studies, for example, through evaluating mentoring programmes.

Our study highlights some key factors for research teams employing lived experience researchers to consider in order to make the lived experience researcher role effective and fulfilling for individuals, rather than tokenistic. This includes actively fostering a culture of empathy within research contexts to enable lived experience researchers to be able to be open and honest about the challenges inherent within the role, be their authentic selves and integrate their lived experience‐based social identity with other important social identities such as that of activist. This will serve the dual purpose of supporting self‐cohesion and personal growth in such researchers and enabling them to make an effective contribution to research.

Careful onboarding for lived experience researchers joining organisations is important to become familiar with the values and objectives of the employing organisation and also clarify role expectations. Setting the scene in supervisory and line management meetings will also be important, for example, taking time to create a relationship within which lived experience researchers feel safe to share concerns or challenges as they arise, confident in the knowledge that this will be empathically received. Self‐reflection on the personal impact of the work, but also how lived experience researchers can use their lived experience to be most impactful, will be important to success (Richmond et al., 2023). Finally, developing and testing staff training designed to address structural stigma and promote a more inclusive, less stigmatising work environment is essential.

In conclusion, to achieve objectives promoted across charities, professional organisations, funding bodies, ethical review panels and journals to increase diversity within research, there is a need to actualise work as a safe space where people feel able to be open and honest about their mental health experiences and periodic struggles without fear of being judged or rejected.

## AUTHOR CONTRIBUTIONS

**Lorna I. Hogg:** Conceptualization; investigation; writing – original draft; methodology; visualization; writing – review and editing; software; formal analysis; project administration; data curation; resources. **Alison Branitsky:** Validation; writing – review and editing; formal analysis. **Anthony P. Morrison:** Conceptualization; validation; writing – review and editing; supervision. **Tim Kurz:** Methodology; validation; writing – review and editing; supervision; conceptualization. **Laura G. E. Smith:** Validation; writing – review and editing; supervision; conceptualization.

## CONFLICT OF INTEREST STATEMENT

All authors declare that they have no conflits of interest.

## Supporting information


Data S1:

## Data Availability

The data that support the findings of this study are available on request from the corresponding author. The data are not publicly available due to privacy or ethical restrictions.
