# Supplementary material for: ‘Lemons to lemonade’: Identity integration in researchers with lived experience of psychosis
Source: Psychol Psychother. 2025 Feb 24;98(3):643–62. doi: 10.1111/papt.12582 (PMC12346258; doi:10.1111/papt.12582)
Supplement: Supplementary file 1 — Data S1: [file PAPT-98-643-s001.docx]

**Supplementary materials**

**Semi-structured interview schedule**

***Introduction***

*I’d like to interview you about your identity as someone who experiences psychosis. The interview should last an hour to an hour and a half. The questions have no ‘right’ or ‘wrong’ answers. I want to hear about your personal experiences and views.*

*To start, I’ll explain what I mean by social identity to make sure you understand this concept. Social identity refers to the way that people's self-concepts are based on their membership in social groups. Examples of social groups include sports teams, religions, nationalities, occupations, sexual orientation, ethnic groups, gender and also health conditions, including mental health conditions such as psychosis. Individuals don’t have to meet regularly with others in the social group to have a social identity and social identities are more than associations with groups but include a felt sense of belonging to the group such that membership of the group influences attitudes and behaviours both to those in the ingroup and also the outgroup. In relation to psychosis, the ingroup would be other people with psychosis and the outgroup possibly those in the general population who do not have psychosis.*

1. **To what extent do you consider yourself to have a social identity based on your experience of psychosis i.e., a feeling of belonging to the group of people who experience psychosis?**

- If not, why does a ‘psychosis social identity’ not resonate for you? Do you feel connected to others who experience psychosis? If so, is this based on having those similar experiences or other factors? If other factors, what are these?

***(If participant has an identity based on their psychotic experiences even if they do not conceptualise this as a ‘Psychosis social identity’, stick with their preferred terminology and adjust rest of questions accordingly)***

1. **How much contact do you have with others in this group? And in what context?**

- What do you feel helps/hinders these relationships forming?

1. **What do you feel are the norms or values associated with this group/identity? How do you personally, and the group generally, enact these norms and values?**
2. **To what extent do you feel a bond, or sense of solidarity, with others in this group?**
3. **What are the positives and negatives of identifying as a member of this group for you?**

- How does this help/hinder your mental health and wellbeing?

1. **What do you as an individual, or the group as a whole, do to manage any negatives associated with this identity?**
2. **How central is being a member of this group to your sense of self i.e. how you see yourself?**
3. **How much do you feel you have in common with others in the group?**
4. **How similar do you feel people in the group are to each other?**
5. **What are other important social identities for you, or other important social groups in your life that you feel a sense of belonging to?**

- How compatible are these with your social identity based on being someone who experiences psychosis?
- How well do you feel your various social identities, including that based on psychosis, integrate into a coherent sense of self?
- What helps/hinders this compatibility/integration?

1. **How does being employed in a leadership position influence how you express your identity as someone who experiences psychosis?**

- Is there a difference between how you express your identity as someone with psychosis in the job and personally? If so, how do you feel about this difference?

1. **Is there anything else that you think is relevant that you would like to contribute?**
2. **Do you have any feedback on the questions I have asked you**?
